# Supplementary material for: MetaRibo-Seq measures translation in microbiomes
Source: Nat Commun. 2020 Jun 29;11:3268. doi: 10.1038/s41467-020-17081-z (PMC7324362; doi:10.1038/s41467-020-17081-z)
Supplement: Supplementary file 10 — Supplementary Data 7 [file 41467_2020_17081_MOESM10_ESM.zip › File2/Confidence_High_Taxonomy/333879_out.krona.html]

Javascript must be enabled to view this page.

members
magnitude
magnitudeUnassigned
count
unassigned
taxon
rank

333879\_out

49

2
49
superkingdom

976
49
phylum

200643
49
class

171549
order
49

family
49
815

genus
49
816

47678

SRS013476\_contig\_number\_24890SRS014287\_contig\_number\_11289SRS014613\_contig\_number\_2099SRS016203\_contig\_number\_9172SRS016335\_contig\_number\_12297SRS017103\_contig\_number\_1801SRS017701\_contig\_number\_5523SRS018049\_contig\_number\_95SRS018133\_contig\_number\_2463SRS018427\_contig\_number\_23433SRS019030\_contig\_number\_1014SRS019178\_contig\_number\_2188SRS019445\_contig\_number\_32036SRS020271\_contig\_number\_2934SRS023583\_contig\_number\_417SRS023715\_contig\_number\_26523SRS024132\_contig\_number\_20810SRS043768\_contig\_number\_19349SRS043841\_contig\_number\_801SRS044535\_contig\_number\_1631SRS045645\_contig\_number\_14790SRS052078\_contig\_number\_204SRS053649\_contig\_number\_3536SRS054905\_contig\_number\_17987SRS058770\_contig\_number\_1261SRS064276\_contig\_number\_20198SRS064757\_contig\_number\_24996SRS065397\_contig\_number\_1503SRS075821\_contig\_number\_4944SRS077335\_contig\_number\_2242SRS077502\_contig\_number\_12929SRS098644\_contig\_number\_23198SRS101268\_contig\_number\_contig-100\_3211.3212SRS1041141\_contig\_number\_4944SRS104975\_contig\_number\_17102SRS105082\_contig\_number\_12396SRS105153\_contig\_number\_5209SRS1054716\_contig\_number\_15568SRS143181\_contig\_number\_contig-100\_2563.79899SRS143523\_contig\_number\_17041SRS144183\_contig\_number\_28412SRS146813\_contig\_number\_25530SRS147346\_contig\_number\_contig-100\_1475.172818SRS147425\_contig\_number\_2967SRS148253\_contig\_number\_8350SRS148817\_contig\_number\_1636SRS149784\_contig\_number\_23134SRS893279\_contig\_number\_11956SRS893295\_contig\_number\_11463
49
species
